# Fine-scale assessment of genetic diversity of trembling aspen in northwestern North America

**DOI:** 10.1186/s12862-016-0810-1

**Published:** 2016-10-26

**Authors:** Mathieu Latutrie, Yves Bergeron, Francine Tremblay

**Affiliations:** 1 Institut de Recherche sur les Forêts, Université du Québec en Abitibi-Témiscamingue, 445 boul. de l’Université, Rouyn-Noranda, QC J9X5E4 Canada; 2 Centre d’Étude de la Forêt, Université du Québec à Montréal, PO Box 8888, Centre-Ville, Montréal, QC H3C3P8 Canada

**Keywords:** Aspen, Beringia, Genetic, Ice-free corridor, Last glacial maximum, Microsatellites, Northwestern North America, Phylogeography

## Abstract

**Background:**

In North America, the last ice age is the most recent event with severe consequences on boreal species’ ranges. Phylogeographic patterns of range expansion in trembling aspen (*Populus tremuloides*) suggested that Beringia is likely to be a refugium and the “ice-free corridor” in Alberta may represent a region where small populations persisted during the last glacial maximum (LGM). The purpose of this study was to ascertain whether the origins of trembling aspen in western North America are reflected in the patterns of neutral genetic diversity and population structure. A total of 28 sites were sampled covering the northwestern part of aspen’s distribution, from Saskatchewan to Alaska. Twelve microsatellite markers were used to describe patterns of genetic diversity. The genetic structure of trembling aspen populations was assessed by using multivariate analyses, Mantel correlograms, neighbor-joining trees and Bayesian analysis.

**Results:**

Microsatellite markers revealed little to no neutral genetic structure of *P. tremuloides* populations in northwestern North America. Low differentiation among populations and small isolation by distance (IBD) were observed. The most probable number of clusters detected by STRUCTURE was K = 3 (∆K = 5.9). The individuals in the populations of the 3 clusters share a common gene pool and showed a high level of admixture. No evidence was found that either Beringia or the “ice-free corridor” were refugia. Highest allelic richness (AR) and lowest heterozygosity (H_o_) were observed in Alberta foothills of the Rocky Mountains.

**Conclusions:**

Contrary to our hypothesis, our results showed that microsatellite markers revealed little to no genetic structure in *P. tremuloides* populations. Consequently, no divergent populations were observed near supposed refugia. The lack of detectable refugia in Beringia and in the “ice-free corridor” was due to high levels of gene flow between trembling apsen populations. More favorable environmental conditions for sexual reproduction and successful trembling aspen seedling establishment may have contributed to increase allelic richness through recombination in populations from the Albertan foothills of the Rocky Mountains.

**Electronic supplementary material:**

The online version of this article (doi:10.1186/s12862-016-0810-1) contains supplementary material, which is available to authorized users.

## Background

Earth has experienced several episodes of severe climatic variation that have led to a succession of ice ages during the Quaternary (2.58 Mya until present) [1]. During this period, many species have experienced a succession of range expansions and contractions that have affected their genetic structure and diversity [1–3]. In their review, Excoffier et al. [2] reported that the effects of these range expansions on genetic diversity could differ markedly from pure demographic expansions. Range expansions are characterized by a decrease in genetic diversity along the expansion axis, owing to recurrent bottlenecks and founder events [4].

In North America, the last ice age is unquestionably the most recent event to have had severe consequences for boreal species genetic diversity [5]. According to estimates by Dyke et al. [6], the Last Glacial Maximum (LGM) occurred 18–21 ky BP. It is therefore considered to be that point when the last massive expansion of plants from different glacial refugia was initiated. Williams [7] and Roberts and Hamann [8] have reconstructed the evolution of land covered by several tree species in North America since the LGM, thereby allowing the identification of potential refugia for those species. Also, molecular studies have shown evidence of genetic structure for several North American plant species [9–14].

Beatty and Provan [10] proposed a set of 10 potential glacial refugia for terrestrial plant and animals in North America. This set was subsequently reduced by Callahan [15] to 6 potential refugia for trembling aspen (*Populus tremuloides* Michaux), including: Beringia, the Grand Banks, the northeastern United States, the “Driftless Area” of the mid-western United States, the “ice-free corridor” along the and eastern slopes of the Alberta Rocky Mountains, e.g., [16–18] and the Clearwater Refugium of northern Idaho, e.g., [19].

Beringia has been suspected of being a refugium for mammals [20], herbaceous plants [10, 21], and trees [11, 22–24] during the last ice age maximum. Simulated suitable habitat during the LGM for some boreal and sub-boreal species such as white spruce (*Picea glauca* [Moench] Voss), black spruce (*Picea mariana* [Mill.] BSP), lodgepole pine (*Pinus contorta* ssp. *latifolia* [Engelm.] Critchfield), and *P. tremuloides* were usually located along the northern Pacific coast and in Beringia, as well as their presence south of the ice sheet [8]. Paleoecological and palynological studies have revealed the presence of *Populus* in Alaska shortly after the beginning of the ice cap melting, suggesting that the genus has persisted in this area [8, 24–26]. For balsam poplar (*Populus balsamifera* L.), recent molecular evidence does not support Alaska as glacial refugium but does confirm the existence of two distinct groups in northwestern North America, i.e., a northern group in Alaska and Yukon, and a central group in central distribution area [12]. Keller et al. [12] concluded that the central group descended from the main demographic refugium of *P. balsamifera* under Pleistocene range restrictions, with an expansion toward its margins during range expansion following LGM. For *P. tremuloides*, two models that were based on paleoecological data suggest the existence of refugial habitats in Beringia and was likely a true refugium for this species. Moreover, Callahan et al. [27] have shown the existence of two distinct groups for trembling aspen, namely, one in southwestern USA, and the other in Canada and Alaska. Within the second group, the higher allelic richness that was detected in aspen populations located in Alaska and in Alberta suggests that Beringia was likely to be a true refugium and that the presence of an “ice-free corridor” in Alberta could have permitted to *P. tremuloides* to persist in this area during the LGM [15].

The existence of an “ice-free corridor” between the Laurentian and Cordilleran ice sheets is debated [16]. Since the ice sheets did not advance at the same time in this region, a temporally and geographically shifting ice-free zone could have existed [20, 28]. The Laurentian and Cordilleran ice sheets only coalesced for a brief span of time [16], while numerous isolated foothills of the Rocky Mountains also could have remained ice-free during the LGM [20, 28], potentially leaving suitable habitats for *P. tremuloides*. Yet, suitable habitat conditions for *P. tremuloides* during the LGM were not found in this area according to the simulations of by Roberts and Hamann [8]. In contrast, Callahan et al. [27] reported a higher level of genetic diversity for *P. tremuloides* in this region. This area appears to be important either as a potentially cryptic refugium or more likely as an admixture zone.

The purpose of this study was to ascertain whether the origin of trembling aspen in western North America is reflected in the patterns of neutral genetic diversity and population structure. In the present study, the glacial origin and post-glacial migration route in the northwestern part of the range was uncovered by studying the area analyzed by Callahan et al. [27] at a finer scale. Our aim was to test whether Beringia and the “ice-free corridor” that was situated between the Laurentian and Cordilleran ice sheets might have been the two glacial refugia for trembling aspen in northwestern North America during the Wisconsin Ice Age. The hypothesis were as follows: 1) aspen populations that were located near refugia (Beringia and the "ice-free corridor") should be highly divergent; 2) within-population diversity should decrease with distance from refugia, due to multiple founder events; 3) the “ice-free corridor” was an admixture zone, where divergent lineages (from the south and from the Alaska) had converged.

## Methods

### Study area and sampling

**Fig. 1:**
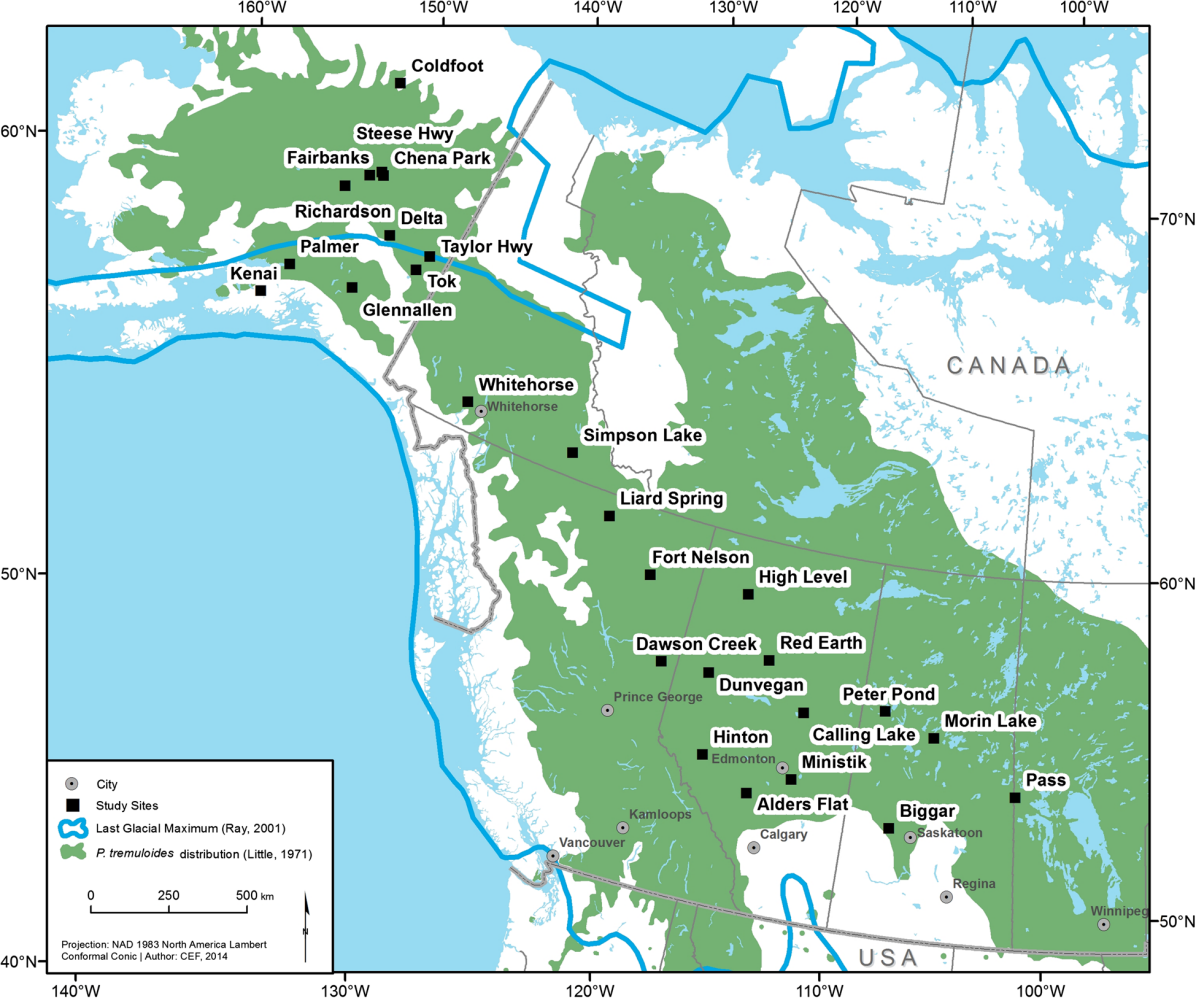
Study populations across the northwestern part of *Populus tremuloides* distribution range

Samples were collected from 28 geo-referenced sampling sites covering the northwestern part of trembling aspen’s distribution from Manitoba to Alaska (51°18′40″ N to 67°25′30″ N; 101°40′37″ W to 150°08′38″ W; Fig. 1), which resulted in a total of 879 trembling aspen trees being sampled. A minimum of 15 trees was sampled from each of 28 sampling sites. We used leaf samples that were collected by a collaborating team from the University of Saskatchewan (Chena Park, Delta, Fairbanks, Richardson, Simpson Lake, Steese Hwy, Taylor Hwy, Tok and Whitehorse; 15 to 45 samples per location) and Utah State University/University of Alaska Fairbanks (Coldfoot, Glennallen, Hinton, Kenai, Liard Spring and Palmer; 30 samples per location). The rest of the samples were collected from root cambia (Alders Flat, Biggar, Calling Lake, Dawson Creek, Dunvegan, Fort Nelson, Glaslyn, High Level, Ministik, Morin Lake, Pass, Peter Pond and Red Earth; 40 samples per location). Within sampling sites, samples were collected to reduce the chance of resampling the same clone. The maximum distances between sampled trees could vary from hundreds of meters to few kilometers. Leaves and root cambia were collected, dried in silica gel, and maintained at room temperature prior to DNA extraction.

### DNA extraction, amplification and sequencing

DNA was extracted using Extract-N-AmpTM Plant kit (Sigma-Aldrich, St Louis, MO, USA) using the manufacturer’s protocol. To evaluate genetic diversity and structure, microsatellite markers were selected because they are rapidly evolving, powerful and economical tools for describing patterns of gene flow and diversity. Samples were amplified at 12 microsatellite loci: PTR1, PTR2, PTR3, PTR4, PTR6, PTR14, PMGC2571, WPMS14, WPMS15, WPMS16, WPMS17, and WPMS20 (Additional file 1: Table S1) [27, 29–32]. Each PCR was performed on a 10 μL total volume: 2 μL of a ten-fold diluted DNA extract (1:10 in ultra pure water); 5 μL QIAGEN® Multiplex PCR Kit (Qiagen, Venlo, Limburg, The Netherlands); and 1 μL H2O and 2 μL primer mix solution at 2 μM, for a final concentration of 0.4 μM. PCR was carried out separately for each primer. Reactions were performed in a Mastercycler® pro Thermal Cyclers (Eppendorf, Hamburg, Germany) with the following protocol: an initial denaturation step at 95 °C for 15 min, followed by 36 cycles of 94 °C for 30 s; a primer-specific annealing temperature for 90 s; 72 °C for 60 s; and a final extension at 60 °C for 30 min. Annealing temperatures were 60 °C for PTR1, PTR2, PTR3, PTR4, WPMS14, WPMS17 and WPMS20 and 63 °C for PTR6, PMGC2571 and WPMS15. For PTR14 and VVPSM16, a touchdown PCR was applied with an annealing temperature ranging from 65 °C to 60 °C during the first ten cycles. PCR products were analyzed on an Automated Capillary DNA Sequencer (ABI 3730, Applied Biosystems, Foster City, CA, USA) using 2 μL of multiplexed PCR products, which were added to 8.4 μL of Hi-Di™ Formamide and 0.11 μL of the GeneScan-500 LIZ size standard (Applied Biosystems). Allele sizes were scored using GENEMAPPER version 5.0 (Applied Biosystems).

### Data manipulation

The original set of 879 samples was then reduced: i) by removing loci with high presence of individuals with three alleles (i.e., > 20 %; that was the case for PTR1 and PTR3); ii) by removing any sample with three alleles at one loci (putative triploid; [33]); and iii) by removing duplicated genotypes (ramets) that were identical at all loci, to keep only one representative of each genotype. We used the software GENODIVE [34] to assign clone identities based on the stepwise mutation model (SMM). In a stepwise mutation model, alleles that differ only by a few repeats in length are thought to be of more recent common ancestry than alleles that differ by many repeats in length [34]. We considered that two individuals belonged to the same clone, if the total genetic distances (mutation frequency between two alleles) for all loci were lower than three mutations, to avoid identifying unique genotypes (genets) that had resulted from scoring errors and soma-clonal mutations (small genetic distances; [35]). Following this operation, the subset contained successively 879, 658 and 526 samples. From the 526 unique genotypes that were isolated from 10 microsatellite markers (without PTR1 and PTR3), populations with less than 7 unique trees were removed (the Glaslyn population) to maintain sufficiently high statistical power for the following analyses. The final dataset consisted of unique diploid genotypes for 10 loci, 523 genets for 27 populations.

### Variation of genetic diversity

All descriptive genetic analyses were carried out with GenAlex v. 6.2 [36]. Allele frequency, allele number, private alleles (defined here as alleles found in a single population) and genetic estimates within populations, including the average number of alleles per locus (N_a_), average number of effective alleles per locus (N_e_), observed heterozygosity (H_o_), and expected heterozygosity (H_e_), were calculated using GenAlex v. 6.2 [36]. To describe genetic diversity within sampling areas across the range, allelic richness (AR) across all ten loci were calculated using FSTAT v. 2.9.3 [37] with rarefaction, a method that was employed to account for differences in sample size. Correlations between AR and H_e_ were computed, while Hardy-Weinberg (HW) equilibrium was assessed by calculating the inbreeding coefficients (F_is_) and their corresponding *P*-values for all sampling sites. We also ran a global test of HW equilibrium for all the samples pooled together. Bonferroni correction was applied when testing the significance of heterozygosity deficit and heterozygosity excess. All of the HW equilibrium tests were performed in FSTAT [37]. Finally, values of AR and H_o_ were interpolated using the inverse distance weighting (IDW) method to create maps of each variable, using ArcMap (Esri, California, USA).

### Genetic structure analyses

Mantel tests and correlograms, together with multivariate analysis of spatial patterns of genetic divergence (PCoA and RDA) were performed with the package *ade4* [38] in the R statistical environment version 2.15.0 [39]. A global simple Mantel test was performed with the function *mantel.rtest* [38] to test for significant correlations between genetic (estimated by F_st_; Additional file 2: Table S2) and geographical distances (in kilometers) between sites, as well as isolation-by-distance patterns. Assumptions of linearity and homoscedasticity were checked before interpreting the results of the Mantel test [40]. The definition of distance classes, both in terms of the total number of classes and their upper and lower limits, is somewhat arbitrary and depends upon the spatial distribution of the populations [40]. A “rule-of-thumb” suggests four to five classes for 20 populations. The Mantel correlogram was constructed by plotting Mantel correlations between the genetic distances for 5 classes of geographical distances with the function *mantel.correlog* in the *vegan* package [41]. Particular care was taken to maintain a constant number of pairs of populations in each class creating unequal distance intervals. To complement the correlogram, we plotted the relationship between genetic and geographical distances, followed by plotting the genetic distance between two sites, which as estimated as Fst /(1-Fst), as a function of geographical distance. We finally performed an analysis of molecular variance (AMOVA) in GenoDive [34]. Pairwise Fst were calculated for each population, together with its corresponding *P*-value, after which Bonferroni corrections were applied (initial *P* = 0.05; number of pairwise tests = 351; adjusted critical *P* = 0.05/351 = 0.00014).

The results of the Mantel test and correlogram were confirmed by multivariate analysis, which was carried out using the functions *dudi.pco* and *pcaiv* to calculate the respective PCoA and RDA ordinations [38]. PCoA was first applied to the Fst matrix (Additional file 2: Table S2), with the retention of the first five axes for further analyses. To analyze patterns in the genetic data, we performed RDA, using scores for the first five axes of the PCoA as the response variables, and longitudinal and latitudinal data as explanatory variables [42]. The output from the RDA, which was obtained with the function *summary*, provided the percentage of the unconstrained variation (PCoA axes representing the genetic differentiation) that was explained by the predictor (geographic location). The function *randtest* was used to evaluate the RDA significance by randomly permuting (Monte-Carlo test) the rows of the explanatory table [38].

To reveal genetic structure, and test whether the samples could be clustered according to their respective distribution zones, we used STRUCTURE v. 2.3.2 software [43]. The analyses were based on an admixture ancestral model. Correlated allele frequencies and a priori sampling locations were used as prior information (LOCPRIOR setting). LOCPRIOR was used to detect any further structures that could not be identified by standard settings [44]. Ten independent runs were performed for each value of K (1–27) with a burn-in of 100 000, followed by 200 000 MCMC iterations. The most likely value of K was determined using the ∆K criterion [45]. STRUCTURE HARVESTER version 0.6.93 was used to extract the results and created a graphical plot of the ∆K criterion [46]. The results were visualized for the best K, with DISTRUCT version 1.1 [47].

Finally, we computed a neighbour-joining tree (NJT) [48] to see how populations are genetically linked to one another, and whether clusters could be isolated similarly to the structuring that was found earlier. The NJT was constructed with POPTREE2 software [49] based on Nei’s standard genetic distance, Ds [50]. The neighbour-joining tree was bootstrapped 1000 times.

### Population genetic bottleneck

M-ratios were estimated to detect historical bottlenecks at each site with the program MPval [51]. To interpret results of the M-ratios, we calculated the critical M-ratio (*Mc*) value for each population with the program M-crit, which was developed by JC Garza and EG Williamson [51]. To calculate *M*_c_ and M-ratios we used a pre-bottleneck value (*θ* = 4 *N*_*e *_μ = 10; *N*_*e*_, the effective population size; the mutation rate, μ) and the parameters were set as recommended by JC Garza and EG Williamson [51]. The settings were: a constant mutation rate (μ), which encompassed a range between 10^−2^ and 10^−6^ mutants/generation/locus; probability of changes greater than one step, *p*_*g*_ = 0.12; and the size of non-one-step changes, Δ_*g*_ =2.8. Each set of simulations consisted of 10 000 iterations with the same values of *θ* for all sites under a two-phase mutation model (TPM). We considered that a M-ratio below the critical value *Mc* was indicative of a population decline. To test for heterozygosity excess, Bottleneck version 1.2.02 [52] was used with a stepwise mutation model (SMM), an infinite allele model (IAM), and a two-phase model (TPM) with 12 % multistep mutations and variance = 0.36 [53]. Mode shifts and heterozygosity excess are transient [54]. To determine which sampling locations had a significant heterozygote excess across loci, a standardized differences test was used. We also used the graphical method to assess bottleneck-induced distortions of allele frequency distributions that cause alleles at low frequency (<0.025) to become less abundant than alleles in one or more intermediate allele frequency classes (e.g. 0.025–0.050) [54]. In this method, the probability (power) of detecting a recent historical bottleneck of fewer than 20 breeding individuals is estimated to be 80 % with eight to ten microsatellite loci [54].

## Results

### Variation of genetic diversity

**Fig. 2:**
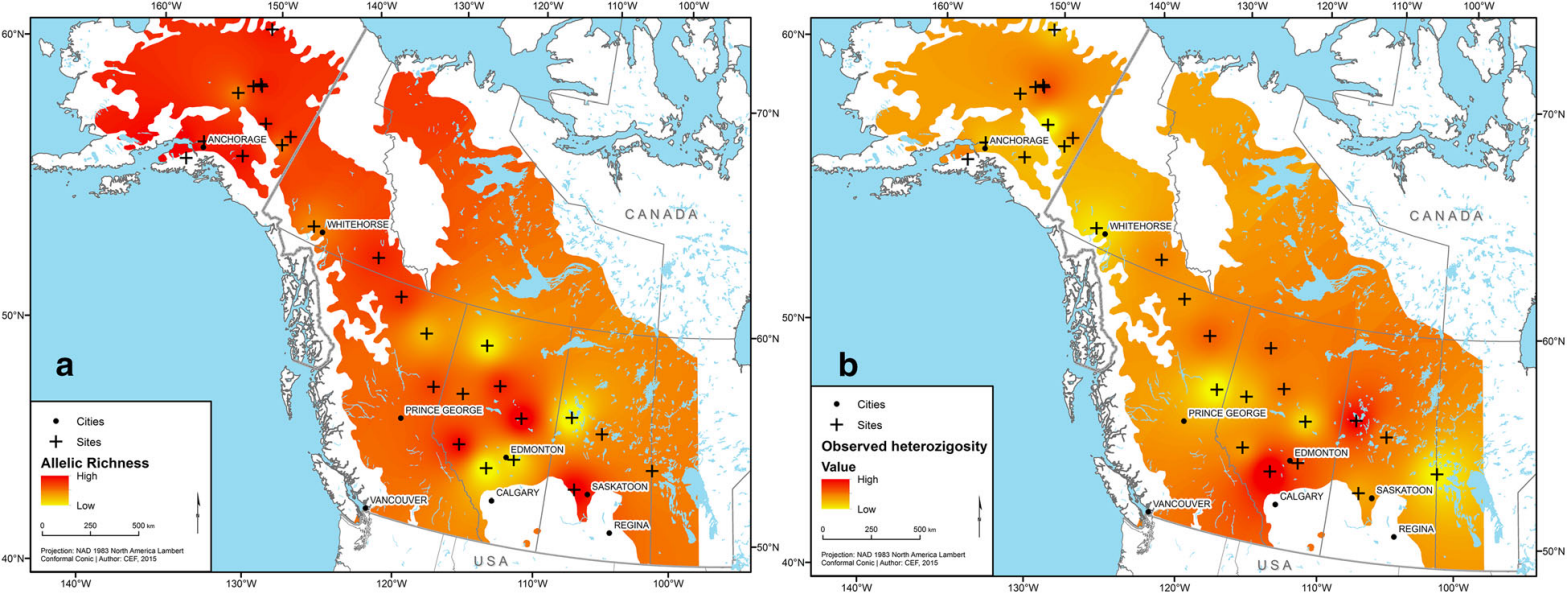
Interpolation of (**a**) allelic richness (AR) and (**b**) observed heterozygosity (H_o_) across the range of *Populus tremuloides* based on average AR and H_o_ values at each sampling site. Across the range, AR and H_o_ respectively varied from 3.94 (Peter Pond) to 5.22 (Kenai) and from 0.542 (Delta) to 0.762 (Alders Flat). *Red* represents areas of higher values and *yellow* represents areas of lower values

Over the entire population, the number of alleles that were observed per locus ranged from 10 (PTR2 and WPMS16) to 30 (PMGC2571; Additional file 1: Table S1). Our results showed that all 12 loci were highly polymorphic and that PTR1 and PTR3 had a high rate of triallelic individuals (Additional file 3: Figure S1). These markers were therefore removed from further analysis. At the population level, AR averaged 4.63 and ranged from 3.94 (Peter Pond) to 5.22 (Kenai). N_a_ ranged from 4.5 (Peter Pond and High Level) to 9.5 (Steese Hwy), with an average of 6.4 (Table 1). The mean N_e_ was 3.5, with lowest value being 2.9 (Richardson, High Level and Dawson Creek) and the highest being 4.3 (Kenai). Across all loci, only 34 individuals had one or more private alleles. Individuals with private alleles were spread across all sampled sites (data not shown). H_o_ had a mean value of 0.629 and was lowest in the Delta population (0.53) and highest in the Alders Flat population (0.76). The mean H_e_ was 0.625, ranging from 0.57 (Pass and Dawson Creek) to 0.66 (Chena Park, Palmer and Red Earth; Table 1). The variation in AR and H_o_ is represented on maps (Fig. 2) to evaluate the spatial genetic variation visually. Higher genetic diversity was observed in Alberta and Alaska for AR, but only in Alberta for H_o_. We found a positive correlation between AR and H_e_ (Pearson product-moment correlation: *r* = 0.61; *P* < 0.001).

**Table 1:** Descriptive genetic composition of 27 *Populus tremuloides* populations in northwestern North America

Population	Latitude	Longitude	N	G	AR^a^	Na	Ne	Ho	He	Fis
Pass	53.603	−101.677	20	9	4.48	4.9 ± 0.69	3.1 ± 0.68	0.58 ± 0.09	0.57 ± 0.07	0.01 ± 0.08
Morin Lake	55.143	−106.072	34	19	4.46	5.8 ± 0.71	3.4 ± 0.56	0.64 ± 0.07	0.65 ± 0.05	0.01 ± 0.1
Biggar	52.315	−107.768	36	18	4.97	7.2 ± 0.94	3.5 ± 0.6	0.62 ± 0.05	0.64 ± 0.05	0.02 ± 0.06
Peter Pond	55.744	−108.776	14	11	3.94	4.5 ± 0.69	3.0 ± 0.52	0.72 ± 0.07	0.61 ± 0.04	−0.2 ± 0.11
Calling Lake	55.292	−112.970	36	26	5.08	8.5 ± 1.28	4.1 ± 1.09	0.58 ± 0.07	0.63 ± 0.07	0.05 ± 0.04
Red Earth	56.607	−115.308	33	29	4.82	7.9 ± 0.98	3.5 ± 0.61	0.64 ± 0.06	0.66 ± 0.05	0.01 ± 0.06
High Level	58.338	−117.237	18	10	4.06	4.5 ± 0.48	2.9 ± 0.36	0.65 ± 0.06	0.6 ± 0.05	−0.1 ± 0.09
Ministik	53.276	−112.932	31	14	4.11	5 ± 0.89	3.2 ± 0.54	0.68 ± 0.09	0.59 ± 0.07	−0.18 ± 0.13
Alders Flat	52.602	−114.964	26	16	3.98	5.2 ± 0.76	2.9 ± 0.36	0.76 ± 0.07	0.61 ± 0.04	−0.26 ± 0.13
Hinton	53.429	−117.531	22	22	4.92	7.6 ± 1.15	3.9 ± 0.92	0.62 ± 0.07	0.64 ± 0.05	0.05 ± 0.05
Dunvegan	55.828	−118.263	33	27	4.56	7.3 ± 0.96	3.5 ± 0.92	0.6 ± 0.06	0.61 ± 0.05	0 ± 0.04
Dawson Creek	55.778	−120.816	8	7	4.7	4.7 ± 0.7	2.9 ± 0.59	0.56 ± 0.07	0.57 ± 0.05	0.03 ± 0.05
Fort Nelson	58.111	−122.746	37	20	4.29	6 ± 1.13	3.4 ± 0.93	0.66 ± 0.06	0.6 ± 0.05	−0.11 ± 0.07
Liard Spring	59.355	−125.957	24	24	4.76	7.3 ± 0.98	3.9 ± 0.83	0.63 ± 0.07	0.65 ± 0.06	0.04 ± 0.03
Simpson Lake	60.676	−129.222	12	11	4.81	5.6 ± 0.64	3.3 ± 0.6	0.64 ± 0.06	0.63 ± 0.05	−0.01 ± 0.06
Whitehorse	60.784	−136.025	17	13	4.46	5.7 ± 0.99	3.3 ± 0.74	0.58 ± 0.07	0.59 ± 0.06	−0.01 ± 0.09
Taylor Hwy	63.887	−142.243	15	15	4.79	6.3 ± 1.04	3.7 ± 0.76	0.63 ± 0.05	0.63 ± 0.06	−0.03 ± 0.07
Tok	63.370	−142.566	26	26	4.51	6.9 ± 0.91	3.6 ± 0.81	0.6 ± 0.05	0.63 ± 0.05	0.05 ± 0.05
Delta	63.809	−145.094	12	12	4.83	5.8 ± 0.95	3.7 ± 0.99	0.53 ± 0.06	0.63 ± 0.05	0.17 ± 0.06
Glennallen	62.005	−145.341	29	29	4.87	7.5 ± 1.16	3.8 ± 0.83	0.61 ± 0.06	0.65 ± 0.06	0.06 ± 0.03
Chena Park	65.105	−147.531	15	15	4.82	5.9 ± 0.69	3.7 ± 0.75	0.71 ± 0.07	0.66 ± 0.05	−0.07 ± 0.09
Steese Hwy	65.161	−147.743	58	56	4.89	9.5 ± 1.27	4.1 ± 1.04	0.65 ± 0.05	0.65 ± 0.05	0 ± 0.04
Fairbanks	64.902	−148.277	9	9	4.67	5.1 ± 0.43	3.3 ± 0.56	0.63 ± 0.05	0.63 ± 0.05	−0.02 ± 0.07
Richardson	64.267	−149.200	10	10	4.47	5.4 ± 0.52	2.9 ± 0.31	0.63 ± 0.05	0.6 ± 0.05	−0.05 ± 0.04
Palmer	61.581	−149.249	27	27	4.81	7.5 ± 1.28	3.9 ± 0.9	0.6 ± 0.06	0.66 ± 0.05	0.08 ± 0.05
Kenai	60.480	−149.724	17	17	5.22	7.3 ± 1.05	4.3 ± 0.96	0.65 ± 0.07	0.65 ± 0.07	−0.01 ± 0.06
Coldfoot	67.425	−150.144	31	31	4.85	8 ± 1.22	3.8 ± 0.91	0.6 ± 0.05	0.65 ± 0.05	0.07 ± 0.04

*N* number of sampled trees genotyped, *G* number of unique genotypes used in the analyses, *AR* allelic richness, *N*_*a*_ average number of alleles per locus, *N*_*e*_ average number of effective alleles per locus, *H*_*o*_ observed heterozygosity, *H*_*e*_ expected heterozygosity, *F*_*is*_ interbreeding coefficient

^a^Calculated with rarefaction method based on the minimum number of unique genotypes

### Patterns of genetic structure

**Fig. 3:**
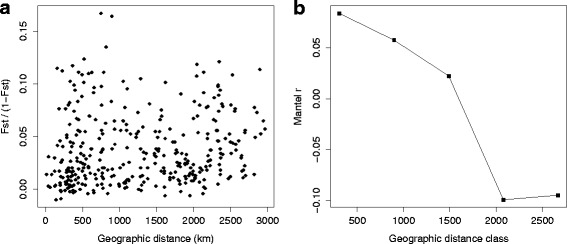
Isolation by distance patterns: **a** Each point represents the genetic distance Fst between two sites as a function of geographical distance (km); **b** Mantel correlogram for 5 geographic distance classes based on F_st_ genetic distances

The genetic and the geographical distance matrices were not strongly correlated (Mantel test r_m_ = 0.134; *P* = 0.024; Fig. 3). The results obtained with the Mantel test and the correlogram were confirmed by RDA (*R* ^*2*^ = 0.092; data not shown). The AMOVA (Table 2) indicated that 3.1 % of the genetic variation (F_st_ = 0.031) was partitioned among populations and 96.9 % within populations (*P* < 0.001). No pairwise differences between populations, as estimated by Fst, appeared to be significant after applying a Bonferroni correction (adjusted critical *P* = 0.00014; Additional file 2: Table S2).

**Table 2:** Results of analysis of molecular variance (AMOVA) for *Populus tremuloides* in northwestern North America (*n* = 523 genets), based on microsatellite allele frequencies

Source of variation	Sum of square	Variance component	% of variance
Within populations	2770.652	5.575	0.969
Among populations	233.945	0.178	0.031
Total	3004.597	5.753	

**Fig. 4:**
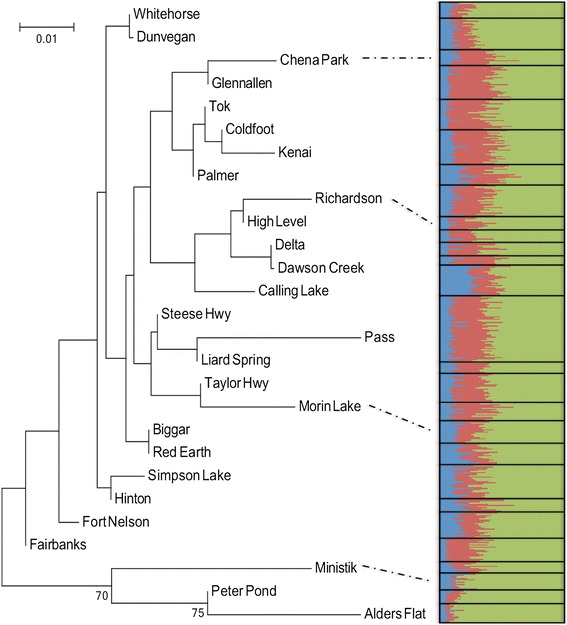
Neighbour-joining tree obtained using Nei’s distance matrix for 27 populations of *Populus tremuloides* with data obtained from 10 polymorphic microsatellite loci. The numbers represent the bootstrap values as percentages. On the right side are the STRUCTURE results graphically displayed to show, for each sample, the probability of belonging to each of the 3 groups detected

Bayesian analysis did not demonstrate the presence of strong population genetic structuring. The most probable number of clusters that were detected by STRUCTURE was K = 3 (ΔK = 5.9) and are displayed in blue, green and red (Fig. 4).

The results of the NJT (Fig. 4), which were based on Nei’s standard genetic distance, were consistent with the results of the Mantel test and RDA, showing low differentiation between sites. Populations that were genetically close to one another are not necessary spatially aggregated. Two clusters can be identified at increased confidence levels (bootstrap values > 50).

### Population genetic bottlenecks

The M-ratio bottleneck test proved to be sensitive to the choice of θ. Significant M-ratio values were obtained for all sites using values of θ = 1 (data not shown). Statistical significance to detected historical bottlenecks was maintained in 16 populations with higher values of θ (10), where M-ratio < *Mc* (Alders Flat, Biggar, Calling Lake, Chena Park, Coldfoot, Fort Nelson, Glennallen, High Level, Liard Spring, Ministik, Morin Lake, Palmer, Red Earth, Simpson Lake, Tok and Whitehorse) [51]. Across all populations and loci, M-ratio varied from 0.583 (High Level) to 0.791 (Steese Hwy; Table 3). Recent bottlenecks with heterozygote excess were detected in six populations (Biggar, Coldfoot, Dawson Creek, Dunvegan, Richardson and Tok; *P* < 0.05), using TPM (Table 3). The graphical method detected recent population bottlenecks in 6 populations (Biggar, Fairbanks, Pass, Morin Lake, Simpson Lake and Tailor Hwy; Additional file 3: Figure S1). Results from the heterozygote excess test and the graphical method were consistent only for 1 population (Biggar).

**Table 3:** Results of bottleneck analyses performed with the software Bottleneck version 1.2.02 (Cornuet and Luikart, [52]) and the program MPval [51] to calculate the M-ratio and M-critical with *θ* = 10 for each of the 27 populations

Population	He	*P*-TPM	*P*-SMM	*P*-IAM	M-Ratio^a^	Mc^b^
Pass	0,607	0,393	0.372	0.436	0,641	0,624
Morin Lake	0,663	0,374	0.058	0.339	**0,615**	**0,696**
Biggar	0,658	**0,014**	**0.002**	0.176	**0,687**	**0,694**
Peter Pond	0,636	0,366	0.613	0.297	0,665	0,647
Calling Lake	0,638	0,058	**0.014**	0.170	**0,684**	**0,723**
Red Earth	0,667	0,063	**0.002**	0.378	**0,698**	**0,73**
High Level	0,632	0,596	0.386	0.304	**0,583**	**0,634**
Ministik	0,617	0,563	0.443	0.272	**0,642**	**0,671**
Alders Flat	0,63	0,183	0.066	0.308	**0,646**	**0,682**
Hinton	0,659	0,175	0.060	0.182	0,748	0,71
Dunvegan	0,618	**0,013**	**0.002**	0.380	0,754	0,722
Dawson Creek	0,611	**0,008**	**0.008**	**0.018**	0,637	0,597
Fort Nelson	0,613	0,386	0.376	0.573	**0,687**	**0,701**
Liard Spring	0,662	0,373	**0.002**	0.629	**0,705**	**0,714**
Simpson Lake	0,658	0,057	**0.012**	0.383	**0,6**	**0,647**
Whitehorse	0,613	0,055	0.058	0.608	**0,614**	**0,664**
Taylor Hwy	0,655	0,057	**0.013**	0.398	0,704	0,676
Tok	0,644	**0,014**	**0.002**	0.355	**0,653**	**0,723**
Delta	0,657	0,063	0.051	0.397	0,716	0,655
Glennallen	0,658	0,056	**0.000**	0.376	**0,723**	**0,73**
Chena Park	0,682	0,631	0.395	0.595	**0,656**	**0,676**
Steese Hwy	0,656	0,059	**0.015**	0.372	0,791	0,767
Fairbanks	0,67	0,167	0.058	0.611	0,663	0,624
Richardson	0,633	**0,017**	**0.002**	0.065	0,682	0,634
Palmer	0,668	0,06	**0.014**	0.365	**0,674**	**0,722**
Kenai	0,674	0,368	0.173	0.382	0,699	0,688
Coldfoot	0,659	**0,002**	**0.000**	0.375	**0,727**	**0,734**

Values in bold show a significant bottleneck detected

For the heterozygosity excess test, we tested a stepwise mutation model, an infinite alleles model and a two-phase mutation model with 12 % multistep mutations and a variance = 0.36. M-ratio average was calculated across loci. Mc is the critical M-value calculated through the M-crit program developed by Garza and Williamson [51]. M-ratio test is significant if M-Ratio < Mc

^a^M-Ratio = number of alleles/range in allele size and range (size of largest allele - size of smallest allele + 1)

^b^Mc is defined such that only 5 % of the simulation values fall below this threshold

## Discussion

The purpose of this study was to ascertain whether the origin of trembling aspen in northwestern North America is reflected in the patterns of genetic diversity and population structure. Contrary to our hypothesis, microsatellite markers revealed little to no genetic structure in *P. tremuloides* populations and indicated little isolation by distance (IBD). Consequently, no divergent populations were observed near supposed refugia suggesting no evidence that Beringia or the “ice-free corridor” were refugia for trembling aspen. Finally, favorable conditions for sexual reproduction and successful trembling aspen seedling establishment could have contributed to the highest AR and lowest H_o_ that were observed in Alberta foothills of the Rocky Mountains.

### Genetic diversity and structure

Our results support the findings of Callahan et al. [27], who found no genetic structuring among populations in the northern part of aspen’s range. Trees are known to have low differentiation at neutral molecular markers, indicating high levels of gene flow among populations [55, 56]. We observed low levels of differentiation and variation in genetic distance between populations (Figs. 3 and  4). Specific aspen traits, such as outbreeding, wind pollination, aeolian seed dispersal, high seed production and/or longevity, can account for this observation. In addition, there was no pronounced pattern of IDB, even at great distances (Fig. 3). We were not able to detect local genetic structure patterns. This indicates that aspen populations in the northern portion of the species range experience high levels of gene flow, making it difficult to identify refugia.

The Rocky Mountains foothills of Alberta, which could have remained ice-free during the last glacial maximum (LGM; [20]), exhibited higher genetic diversity (AR), which is consistent with the observations of Callahan et al. [27]. Moreover, no divergent lineages or specific private alleles were found in this area or north of this region in Beringia. The lack of detectable refugia in Beringia and in the “ice-free corridor” was due to high levels of gene flow between trembling apsen populations. We agree that the Alberta foothills were not an area of admixture because we don’t have highly differentiated populations. More favorable environmental conditions for sexual reproduction and successful trembling aspen seedling establishment in this area [57, 58] may have contributed to increase allelic richness through recombination in populations from the Albertan foothills of the Rocky Mountains. The existence of a refugium in Beringia during the LGM has been reported for *P. glauca* [9, 23] and was consistent with the model simulation of suitable refugial habitats for this species (performed with the Community Climate Model and the Geophysical Fluid Dynamics Laboratory Model) [8], which suggested the presence of *P. glauca* in Beringia during the LMG. Moreover, those simulations also suggested the presence of suitable habitats for *P. contorta* and *P. tremuloides* in Beringia [8]. For the closely related species *P. balsamifera*, recent molecular evidence did not support Beringia as a glacial refugium, but confirmed the existence of two distinct clusters in our sample area of northwestern North America [12].

### Population genetic bottlenecks

The M-ratio bottleneck test proved to be sensitive to the choice of θ, with significant M-ratio values being obtained for all sites using values of θ = 1. The M-ratio detected persistent bottleneck signatures in 16 populations with θ = 10. Low M-ratios that were detected indicate that these 16 populations might have suffered from demographic declines, although they were not severely reduced in their genetic potential. We did not detect strong evidence for excess heterozygosity. Consistent results were obtained only for 1 population. Indeed, wild populations are rarely completely closed and even small numbers of migrants can mask the genetic signature of bottlenecks [59, 60]. Under TPM, the populations that were subject to a recent reduction in size (Biggar, Coldfoot, Dawson Creek, Dunvegan, Richardson and Tok) were not spatially clustered and were present all over the sampled territory without showing any sign of spatial structure. Large effective population size implies that polymorphisms can persist during extended periods of time [56], even during reduction of the species distributional range. At low effective population sizes, asexual reproduction might better preserve heterozygosity than outcrossing at least in the short-term [56], hereby masking recent reductions in population size.

## Conclusion

Most of the studies that have detected phylogeographic patterns in boreal tree species in western North America (reviewed by [13]) have used uniparental inherited cpDNA [23], mtDNA markers [61], or more recently genomic data (e.g., SNPs) [12]. For *P. glauca*, LL Anderson, FS Hu and KN Paige [9] suggested that the greater relative rate of mutation of nuclear microsatellites may allow finer scale resolution of the historic dynamics of populations (including the number, location, and population sizes of refugia), compared to chloroplast DNA that have extremely slow mutation rates (estimated to be 5.3 × 10^−9^ mutations per gene per generation). Their results with nuclear microsatellite markers support the idea that north-central Alaska served as a glacial refugium during the last glacial maximum for white spruce. Three genetic groups were detected: the first consisted of one population from north-central Alaska (the northern-most population sampled in Alaska, Dalton Highway); the second with one population from southern Manitoba; and the last group included the remaining 20 populations (ranging from Wisconsin and continuing a northwestwardly fashion into southern and central Alaska) forming the last group. These results revealed that there is not much structure and differentiation to be found for this boreal species, a result similar to what we found in trembling aspen. For *P. tremuloides*, Callahan et al. [27] found 2 distinct groups, with significant correlation of genetic and geographical distances and low AR, solely in the southwestern USA, but nothing in Beringia. Our study did not find any structuring in northwestern North America. The historic dynamics of the populations vary from one species to another. In conclusion, future studies should combine different approaches and molecular analyses to elucidate the glacial origin and post-glacial migration route in the northwestern part of the species’ range.

## Additional files

Additional file 1: Table S1. Primer sequences, size range (in base pairs; bp) and number of alleles observed for 12 microsatellite loci of *Populus tremuloides.* (DOC 44 kb)
Additional file 2: Table S2. Table of pairwise F_st_ and corresponding differentiation for the 27 populations sampled obtained with Genodive (Meirmans and Van Tienderen, [34]). The lower diagonal represents the Fst values and the upper diagonal represents the associated *P*-values obtained with 1000 iterations. **represents the significant differences following Bonferoni correction (adjusted critical *P* = 0.00014) and ns means “not significant”. (DOC 111 kb)
Additional file 3: Figure S1. Allele frequency distribution for the 6 populations that experienced bottlenecks. Histograms were created based on 10 microsatellite loci. The Y-axis represents the number of alleles per class of frequency. (TIF 6077 kb)
