# Supplementary material for: Fine-scale assessment of genetic diversity of trembling aspen in northwestern North America
Source: BMC Evol Biol. 2016 Oct 26;16:231. doi: 10.1186/s12862-016-0810-1 (PMC5080688; doi:10.1186/s12862-016-0810-1)
Supplement: Additional file 1: Table S1. — Primer sequences, size range (in base pairs; bp) and number of alleles observed for 12 microsatellite loci of Populus tremuloides. (DOC 44 kb) [file 12862_2016_810_MOESM1_ESM.doc]

Additional file 1: Table S1. Primer sequences, size range (in base pairs; bp) and number of alleles observed for 12 microsatellite loci of *Populus tremuloides*

| Locus | Repeat | Primer sequence (5’→3’) | Dye colour | Size range (bp) | Number of alleles | Source |
| --- | --- | --- | --- | --- | --- | --- |
| PTR1 | (GGT)n (AGG)n | AGCGCGTGCGGATTGCCATT (F)  TTAGTTTCCCGTCACCTCCTGTTAT (R) | FAM | 239-278 | 12 | Dayanandan et al., 1998 |
| PTR2 | (TGG)n | AAGAAGAACTCGAAGATGAAGAACT (F)  ACTGACAAAACCCCTAATCTAACAA (R) | VIC | 201-229 | 10 | Dayanandan et al., 1998 |
| PTR3 | (TC)n | CACTCGTGTTGTCCTTTTCTTTTCT (F)  AGGATCCCTTCCCTTTAGTAT (R) | NED | 184-274 | 26 | Dayanandan et al., 1998 |
| PTR4 | (TC)n | AATGTCGAGGCCTTTCTAAATGTCT (F)  GCTTGAGCAACAAACACACCAGATG (R) | PET | 196-236 | 18 | Dayanandan et al., 1998 |
| PTR6 | (AT)n | AGAAAAGCAGATTGAGAAAAGAC (F)  CTAGTATAGAGAAAGAAGAAGCAGAAA (R) | VIC | 184-217 | 12 | Rahman et al., 2000 |
| PTR14 | (TGG)n | TCCGTTTTTGCATCTCAAGAATCAC (F)  ATACTCGCTTTATAACACCATTGTC (R) | NED | 131-200 | 18 | Rahman et al., 2000 |
| WPMS14 | (CGT)n | CAGCCGCAGCCACTGAGAAATC (F)  GCCTGCTGAGAAGACTGCCTTGAC (R) | PET | 198-252 | 20 | Smulders et al., 2001 |
| WPMS15 | (CCT)n | CAACAAACCATCAATGAAGAAGAC (F)  AGAGGGTGTTGGGGGTGACTA (R) | VIC | 181-211 | 11 | Smulders et al., 2001 |
| WPMS16 | (GTC)n | CTCGTACTATTTCCGATGATGACC (F)  AGATTATTAGGTGGGCCAAGGACT (R) | FAM | 148-203 | 10 | Smulders et al., 2001 |
| WPMS17 | (CAC)n | ACATCCGCCAATGCTTCGGTGTTT (F)  GTGACGGTGGTGGCGGATTTTCTT (R) | NED | 115-157 | 14 | Smulders et al., 2001 |
| WPMS20 | (TTCTGG)n | GTGCGCACATCTATGACTATCG (F)  ATCTTGTAATTCTCCGGGCATCT (R) | VIC | 210-240 | 11 | Smulders et al., 2001 |
| PMGC2571 | (GA)n | TCTCGCAGATTCATGTAACCC (F)  GACTGTATGTTGACCATGCCC (R) | PET | 86-149 | 30 | Tuskan et al., 2006 |

Dye colours acronyms: FAM, Blue; VIC, Green; NED,Yellow; PET, Red
