# Supplementary material for: Fine-scale assessment of genetic diversity of trembling aspen in northwestern North America
Source: BMC Evol Biol. 2016 Oct 26;16:231. doi: 10.1186/s12862-016-0810-1 (PMC5080688; doi:10.1186/s12862-016-0810-1)
Supplement: Additional file 2: Table S2. — Table of pairwise Fst and corresponding differentiation for the 27 populations sampled obtained with Genodive (Meirmans and Van Tienderen, [34]). The lower diagonal represents the Fst values and the upper diagonal represents the associated P-values obtained with 1000 iterations. **represents the significant differences following Bonferoni correction (adjusted critical P = 0.00014) and ns means “not significant”. (DOC 111 kb) [file 12862_2016_810_MOESM2_ESM.doc]

|  | **1** | **2** | **3** | **4** | **5** | **6** | **7** | **8** | **9** | **10** | **11** | **12** | **13** | **14** | **15** | **16** | **17** | **18** | **19** | **20** | **21** | **22** | **23** | **24** | **25** | **26** | **27** |
| --- | --- | --- | --- | --- | --- | --- | --- | --- | --- | --- | --- | --- | --- | --- | --- | --- | --- | --- | --- | --- | --- | --- | --- | --- | --- | --- | --- |
| **1 - Pass** | -- | ** | ns | ** | ns | ns | ns | ** | ** | ns | ns | ns | ns | ns | ns | ns | ns | ns | ns | ns | ns | ns | ns | ns | ns | ns | ns |
| **2 - Morin Lake** | 0.093 | -- | ns | ** | ** | ** | ns | ** | ** | ** | ns | ns | ** | ns | ns | ns | ns | ** | ns | ** | ** | ** | ** | ** | ** | ** | ** |
| **3 - Biggar** | 0.077 | 0.043 | -- | ** | ns | ns | ns | ** | ** | ns | ns | ns | ns | ns | ns | ns | ns | ns | ns | ns | ns | ns | ns | ns | ns | ns | ns |
| **4 - Peter Pond** | 0.092 | 0.071 | 0.069 | -- | ** | ** | ns | ** | ns | ns | ** | ** | ** | ** | ** | ns | ** | ** | ns | ** | ns | ** | ns | ** | ** | ns | ** |
| **5 - Calling Lake** | 0.074 | 0.053 | 0.033 | 0.071 | -- | ** | ns | ** | ** | ns | ns | ns | ns | ns | ns | ns | ns | ** | ns | ns | ns | ns | ns | ns | ns | ns | ns |
| **6- Red Earth** | 0.061 | 0.031 | 0.010 | 0.049 | 0.025 | -- | ns | ** | ** | ns | ns | ns | ns | ns | ns | ns | ns | ns | ns | ns | ns | ns | ns | ns | ns | ns | ns |
| **7 - High Level** | 0.087 | 0.050 | 0.037 | 0.065 | 0.026 | 0.027 | -- | ** | ** | ns | ns | ns | ns | ns | ns | ns | ns | ns | ns | ns | ns | ns | ns | ns | ns | ns | ns |
| **8 - Ministik** | 0.143 | 0.098 | 0.105 | 0.090 | 0.101 | 0.086 | 0.100 | -- | ** | ** | ** | ** | ** | ** | ** | ** | ** | ** | ns | ** | ** | ** | ** | ns | ** | ** | ** |
| **9 - Alders Flat** | 0.141 | 0.084 | 0.081 | 0.052 | 0.076 | 0.062 | 0.088 | 0.103 | -- | ** | ** | ** | ** | ** | ** | ns | ** | ** | ** | ** | ** | ** | ns | ** | ** | ** | ** |
| **10 - Hinton** | 0.061 | 0.036 | 0.014 | 0.037 | 0.013 | 0.004 | 0.028 | 0.074 | 0.044 | -- | ns | ns | ns | ns | ns | ns | ns | ns | ns | ns | ns | ns | ns | ns | ns | ns | ns |
| **11 - Dunvegan** | 0.053 | 0.037 | 0.020 | 0.065 | 0.034 | 0.012 | 0.047 | 0.100 | 0.063 | 0.007 | -- | ns | ns | ns | ns | ns | ns | ns | ns | ns | ns | ns | ns | ns | ns | ns | ns |
| **12 - Dawson Creek** | 0.052 | 0.054 | 0.041 | 0.089 | 0.017 | 0.039 | 0.020 | 0.099 | 0.110 | 0.026 | 0.040 | -- | ns | ns | ns | ns | ns | ns | ns | ns | ns | ns | ns | ns | ns | ns | ns |
| **13 - Fort Nelson** | 0.075 | 0.072 | 0.050 | 0.077 | 0.062 | 0.039 | 0.058 | 0.119 | 0.100 | 0.037 | 0.042 | 0.049 | -- | ns | ns | ns | ns | ns | ns | ns | ns | ns | ns | ns | ns | ns | ** |
| **14- Liard Spring** | 0.046 | 0.037 | 0.020 | 0.038 | 0.019 | 0.004 | 0.031 | 0.093 | 0.053 | -0.004 | 0.015 | 0.042 | 0.039 | -- | ns | ns | ns | ns | ns | ns | ns | ns | ns | ns | ns | ns | ns |
| **15 - Simpson Lake** | 0.083 | 0.037 | 0.017 | 0.056 | 0.025 | 0.013 | 0.046 | 0.095 | 0.068 | 0.001 | 0.018 | 0.027 | 0.052 | 0.011 | -- | ns | ns | ns | ns | ns | ns | ns | ns | ns | ns | ns | ns |
| **16 - Whitehorse** | 0.073 | 0.049 | 0.015 | 0.054 | 0.052 | 0.020 | 0.058 | 0.092 | 0.065 | 0.010 | 0.012 | 0.061 | 0.059 | 0.020 | 0.017 | -- | ns | ns | ns | ns | ns | ns | ns | ns | ns | ns | ns |
| **17 - Taylor Hwy** | 0.069 | 0.027 | 0.018 | 0.065 | 0.012 | 0.009 | 0.033 | 0.106 | 0.077 | -0.002 | 0.015 | 0.030 | 0.050 | 0.003 | 0.012 | 0.025 | -- | ns | ns | ns | ns | ns | ns | ns | ns | ns | ns |
| **18 - Tok** | 0.071 | 0.036 | 0.014 | 0.051 | 0.024 | 0.011 | 0.031 | 0.097 | 0.063 | 0.005 | 0.020 | 0.024 | 0.047 | 0.005 | 0.005 | 0.019 | 0.002 | -- | ns | ns | ns | ns | ns | ns | ns | ns | ns |
| **19 - Delta** | 0.051 | 0.029 | 0.012 | 0.050 | 0.010 | 0.005 | 0.002 | 0.072 | 0.084 | -0.006 | 0.011 | -0.006 | 0.033 | 0.008 | 0.012 | 0.021 | -0.010 | 0.004 | -- | ns | ns | ns | ns | ns | ns | ns | ns |
| **20 - Glennallen** | 0.053 | 0.033 | 0.010 | 0.046 | 0.023 | 0.009 | 0.031 | 0.100 | 0.074 | 0.006 | 0.015 | 0.017 | 0.034 | 0.002 | 0.004 | 0.020 | -0.001 | -0.002 | -0.001 | -- | ns | ns | ns | ns | ns | ns | ns |
| **21 - Chena Park** | 0.072 | 0.043 | 0.029 | 0.044 | 0.021 | 0.020 | 0.043 | 0.090 | 0.061 | 0.018 | 0.028 | 0.025 | 0.047 | 0.012 | 0.023 | 0.053 | 0.018 | 0.009 | 0.017 | 0.003 | -- | ns | ns | ns | ns | ns | ns |
| **22 - Steese Hwy** | 0.047 | 0.033 | 0.017 | 0.044 | 0.018 | 0.008 | 0.033 | 0.082 | 0.053 | -0.004 | 0.010 | 0.022 | 0.037 | -0.001 | 0.006 | 0.015 | 0.001 | 0.002 | 0.004 | -0.001 | 0.014 | -- | ns | ns | ns | ns | ns |
| **23 - Fairbanks** | 0.068 | 0.042 | 0.018 | 0.037 | 0.031 | 0.014 | 0.034 | 0.086 | 0.053 | 0.002 | 0.024 | 0.008 | 0.052 | 0.013 | 0.007 | 0.020 | 0.020 | 0.004 | 0.006 | 0.003 | 0.030 | 0.003 | -- | ns | ns | ns | ns |
| **24 - Richardson** | 0.102 | 0.041 | 0.013 | 0.073 | 0.036 | 0.011 | 0.024 | 0.075 | 0.069 | 0.001 | 0.013 | 0.021 | 0.070 | 0.028 | 0.009 | 0.010 | 0.012 | 0.008 | -0.009 | 0.017 | 0.029 | 0.017 | 0.014 | -- | ns | ns | ns |
| **25 - Palmer** | 0.050 | 0.036 | 0.011 | 0.032 | 0.026 | 0.008 | 0.022 | 0.089 | 0.062 | 0.007 | 0.023 | 0.016 | 0.035 | 0.004 | 0.006 | 0.018 | 0.006 | -0.002 | -0.002 | -0.002 | 0.014 | 0.009 | -0.003 | 0.012 | -- | ns | ns |
| **26 - Kenai** | 0.061 | 0.044 | 0.022 | 0.046 | 0.022 | 0.013 | 0.033 | 0.108 | 0.080 | 0.016 | 0.032 | 0.036 | 0.062 | 0.007 | 0.009 | 0.034 | 0.010 | 0.005 | 0.011 | 0.005 | 0.023 | 0.013 | 0.024 | 0.029 | 0.003 | -- | ns |
| **27- Coldfoot** | 0.054 | 0.031 | 0.014 | 0.050 | 0.023 | 0.009 | 0.026 | 0.098 | 0.074 | 0.005 | 0.020 | 0.017 | 0.043 | 0.003 | 0.000 | 0.020 | 0.004 | -0.004 | -0.000 | -0.001 | 0.016 | 0.005 | 0.008 | 0.016 | -0.003 | 0.001 | -- |

Additional file 2: Table S2. Table of pairwise F_st_ and corresponding differentiation for the 27 populations sampled obtained with Genodive (Meirmans and Van Tienderen, 2004). The lower diagonal represents the Fst values and the upper diagonal represents the associated *P*-values obtained with 1000 iterations. ** represents the significant differences following Bonferoni correction (adjusted critical *P* = 0.00014) and ns means “not significant”.
