# Supplementary figures and images for: Fine-scale assessment of genetic diversity of trembling aspen in northwestern North America
Source: BMC Evol Biol. 2016 Oct 26;16:231. doi: 10.1186/s12862-016-0810-1 (PMC5080688; doi:10.1186/s12862-016-0810-1)

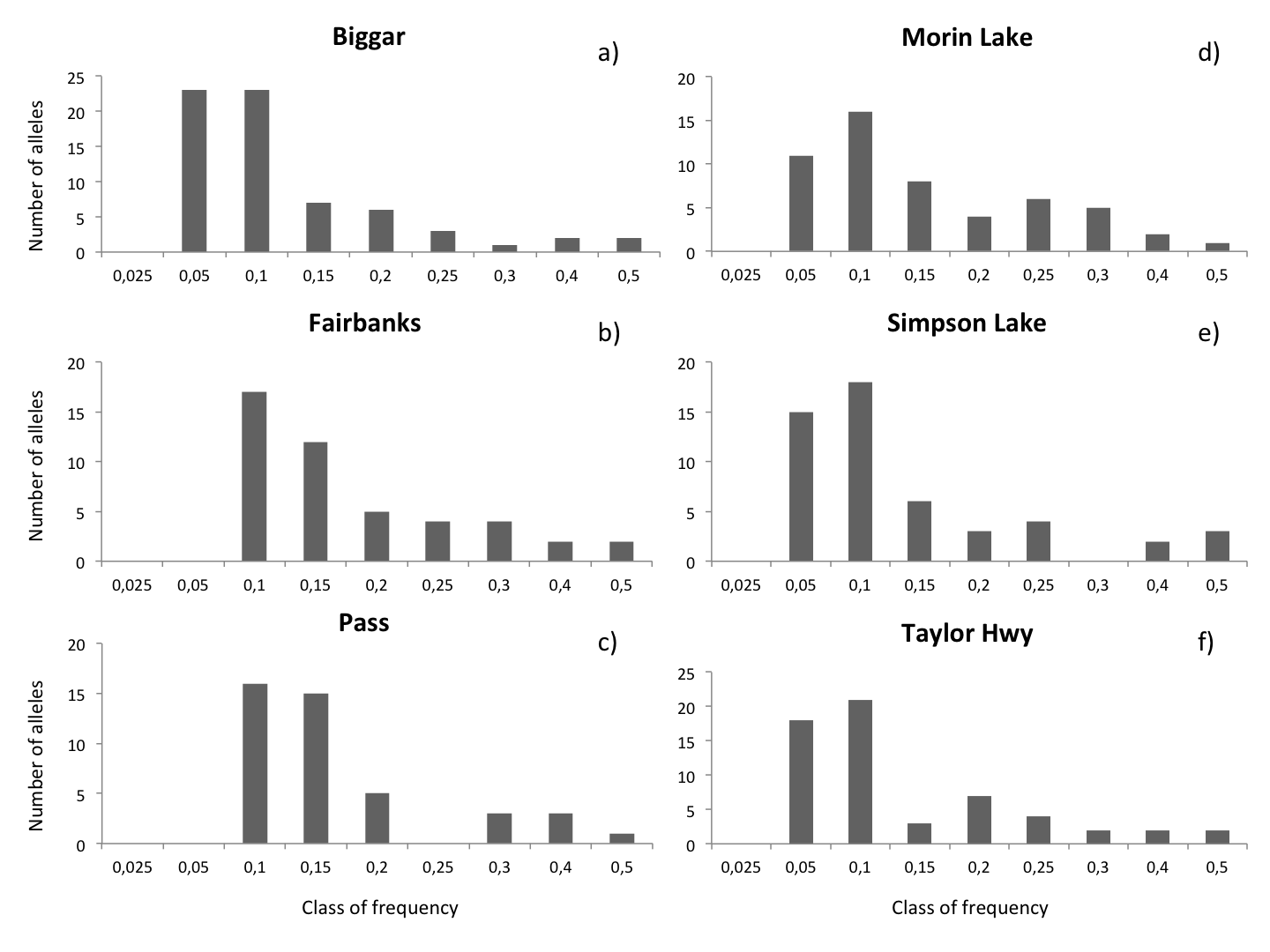

Supplement: Additional file 3: Figure S1. — Allele frequency distribution for the 6 populations that experienced bottlenecks. Histograms were created based on 10 microsatellite loci. The Y-axis represents the number of alleles per class of frequency. (TIF 6077 kb) [file 12862_2016_810_MOESM3_ESM.tif]
